# Green Tea Polyphenols Reduce Body Weight in Rats by Modulating Obesity-Related Genes

**DOI:** 10.1371/journal.pone.0038332

**Published:** 2012-06-08

**Authors:** Chuanwen Lu, Wenbin Zhu, Chwan-Li Shen, Weimin Gao

**Affiliations:** 1 Department of Environmental Toxicology, The Institute of Environmental and Human Health, Texas Tech University, Texas Tech University Health Sciences Center, Lubbock, Texas, United States of America; 2 Department of Pathology, Texas Tech University Health Sciences Center, Lubbock, Texas, United States of America; State University of Rio de Janeiro, Biomedical Center, Institute of Biology, Brazil

## Abstract

Beneficial effects of green tea polyphenols (GTP) against obesity have been reported, however, the mechanism of this protection is not clear. Therefore, the objective of this study was to identify GTP-targeted genes in obesity using the high-fat-diet-induced obese rat model. A total of three groups (n = 12/group) of Sprague Dawley (SD) female rats were tested, including the control group (rats fed with low-fat diet), the HF group (rats fed with high-fat diet), and the HF+GTP group (rats fed with high-fat diet and GTP in drinking water). The HF group increased body weight as compared to the control group. Supplementation of GTP in the drinking water in the HF+GTP group reduced body weight as compared to the HF group. RNA from liver samples was extracted for gene expression analysis. A total of eighty-four genes related to obesity were analyzed using PCR array. Compared to the rats in the control group, the rats in the HF group had the expression levels of 12 genes with significant changes, including 3 orexigenic genes (Agrp, Ghrl, and Nr3c1); 7 anorectic genes (Apoa4, Cntf, Ghr, IL-1β, Ins1, Lepr, and Sort); and 2 genes that relate to energy expenditure (Adcyap1r1 and Adrb1). Intriguingly, the HF+GTP group restored the expression levels of these genes in the high-fat-induced obese rats. The protein expression levels of IL-1β and IL-6 in the serum samples from the control, HF, and HF+GTP groups confirmed the results of gene expression. Furthermore, the protein expression levels of superoxide dismutase-1 (SOD1) and catechol-O-methyltransferase (COMT) also showed GTP-regulated protective changes in this obese rat model. Collectively, this study revealed the beneficial effects of GTP on body weight via regulating obesity-related genes, anti-inflammation, anti-oxidant capacity, and estrogen-related actions in high-fat-induced obese rats.

## Introduction

Over two-thirds of the adults in the United States are overweight or obese and over one-third of U.S. adults are obese [1]. The prevalence of obesity is rising rapidly in many countries, so obesity is seen as a global pandemic. The consequences of this are not only the social and psychological effects of excessive weight, but also the significant morbidity and premature mortality associated with the serious medical conditions that obesity predisposes to, including type II diabetes, hypertension, coronary artery disease, and various forms of cancer [2], [3].

Many risk factors, such as increased caloric intake, reduced energy expenditure, energy balance influenced by the central nervous system, adaptive thermogenesis, neuropeptides, and neurotransmitters, have been recognized to contribute to obesity [4]. The role of the interactions between environmental and genetic factors in the contribution to complex polygenic obesity and common obesity has been more important as no efficient treatment, apart from major surgery, currently exists [5]. Therefore, by the discovery of novel genes or new etiological pathways, innovative therapies, preventive measures, and pharmacogenetical strategies can be found and/or used in obesity studies.

Green tea is one of the most popular beverages in the world. The impacts of green tea consumption on weight loss have been reported in clinical [6], [7], [8], [9], [10], [11], [12] and laboratory animal studies [13]. Such an anti-obesity effect of green tea is probably due to its capacity in elevating thermogenesis and fat oxidation, lowering lipid peroxidation [14], [15], as well as suppressing appetite and nutrient absorption [16]. Our previous studies suggest the positive impacts of green tea polyphenols (GTP, extracts of green tea) could be from its ability in suppressing chronic inflammation and oxidative stress damage, increasing antioxidant capacities, or estrogen-related effect [17], [18], [19], [20], [21]. However, the effects of GTP on obesity along with its related mechanism(s) are not clear. We hypothesize that supplementation of GTP reduces body weight by regulating obesity related genes; and such changes are from down-regulating orexigenic genes, stimulating anorectic genes, increasing energy expenditure, suppressing proinflammatory cytokine production, and/or elevating antioxidant capacity. Therefore, the present study was designed to not only establish a reliable high-fat-induced rat model but investigate the potential benefits of GTP on body weight in these obese rats. Furthermore, the molecular mechanisms including GTP-targeted genes in regulating obesity, anti-inflammatory, anti-oxidant capacities, and estrogen-associated effects were also evaluated to advance the understanding of beneficial effects of GTP on overweight and/or obesity.

## Materials and Methods

### Animals and GTP Treatments

Virgin Sprague Dawley (SD) female rats (n = 36, 3-month old, Harlan Laboratories, Indianapolis, IN, USA) were housed in an environmentally controlled animal care facility and acclimated for 5 days on an AIN-93M diet and distilled water ad libitum prior to the start of experiments. The diets were based on a modification of the AIN-93M diet and supplied by Research Diets Inc. (New Brunswick, NJ, USA). The same amount of minerals and vitamins were given to all animals.

Rats were randomized by weight into two groups: 1) control (a low-fat diet, 10% energy as fat, n = 12); or 2) a high-fat diet (HF, 45% energy as fat, n = 24) for 4 months. Animals in the control group continued on a low-fat diet for an additional 4 months (the control group). Animals in the HF diet group were randomly divided into two treatments, with (the HF+GTP group, n = 12) or without GTP (the HF group, n = 12) in drinking water, in addition to an HF diet for another 4 months. Daily food consumption was recorded. Weekly body weight was also recorded. Rats in the HF+GTP group had free access to distilled water containing 0.5% (wt/vol) GTP. GTP was purchased from the same source as that used in our previous studies (Shili Natural Product Company, Inc., Guangxi, China) with purity higher than 98.5% [17], [18], [19], [20], [21]. Every 1000 mg GTP contained 464 mg (-)-epigallocatechin gallate, 112 mg (-)-epicatechin gallate, 100 mg (-)-epicatechin, 78 mg (-)-epigallocatechin, 96 mg (-)-gallocatechin gallate, and 44 mg catechin according to high-performance liquid chromatography analysis. In our previous study, we provided the same concentration of GTP (0.5%, wt/vol) in the drinking water to female rats for 20 weeks and found these was no liver damage to the studied animals as assessed by liver enzyme activity in serum [22]. Rats were kept in individual stainless steel cages under a controlled temperature of 21±2°C with a 12 h light-dark cycle. All procedures were approved by the Texas Tech University Health Sciences Center Institutional Animal Care and Use Committee.

### Isolation of Total RNA

Rats (11 months old) were anesthetized and euthanized (not fasted), and liver samples were collected at end of the experiment. Total RNA was isolated from liver samples from the control, HF, and HF+GTP groups using an RNeasy® plus mini kit (Qiagen, Valencia, CA) following the manufacturer’s protocol as described in our previous study [23]. Briefly, the lysate liver sample was homogenized, genomic DNA removed, and purified. The RNA was eluted by 30 µL RNase-free water and stored at −80°C until use. The total extracted RNA was determined by measuring OD at 260 nm using a Nano-Drop 1000 Spectrophotometer (Thermo Scientific, Waltham, MA). The quality and purity of total RNA were evaluated by agarose gel electrophoresis using Horizon 11.14 (Life Technologies, Gaithersburg, MD). Total RNA samples used for RT–PCR experiments had good integrity and had OD A260/A280 ratios between 1.99–2.08 and concentration ≥45 µg/mL. The total RNA samples were further treated by DNA-free™ DNase (Applied Biosystems, Austin, TX) to remove possible DNA contamination. Three replicates, each of which were pooled sample from 3 individual rats in each group (9 rats total), were analyzed for each of the control, HF, and HF+GTP groups.

### Reverse Transcription-PCR

cDNA was prepared using an RT^2^ PCR array first strand kit (SABiosciences Corporation, Frederick, MD) according to manufacturer’s instructions. Briefly, 1 µg of total RNA was mixed with 2 µL of 5× gDNA elimination buffer to form a total volume of 10 µL genomic DNA elimination mixture with RNase-free H_2_O, incubated at 42°C for 5 min, and chilled on ice immediately. The mixture was then incubated with a 10 µL RT Cocktail at 42°C for 15 min, and the reaction stopped by heating at 95°C for 5 min to inactivate the reverse transcriptase. The 20 µL cDNA synthesis reaction mixture was diluted to 111 µL by adding 91 µL RNase-free H_2_O and kept on ice for further use.

### Array-based SYBR® Green RT-PCR

**Figure 1:**
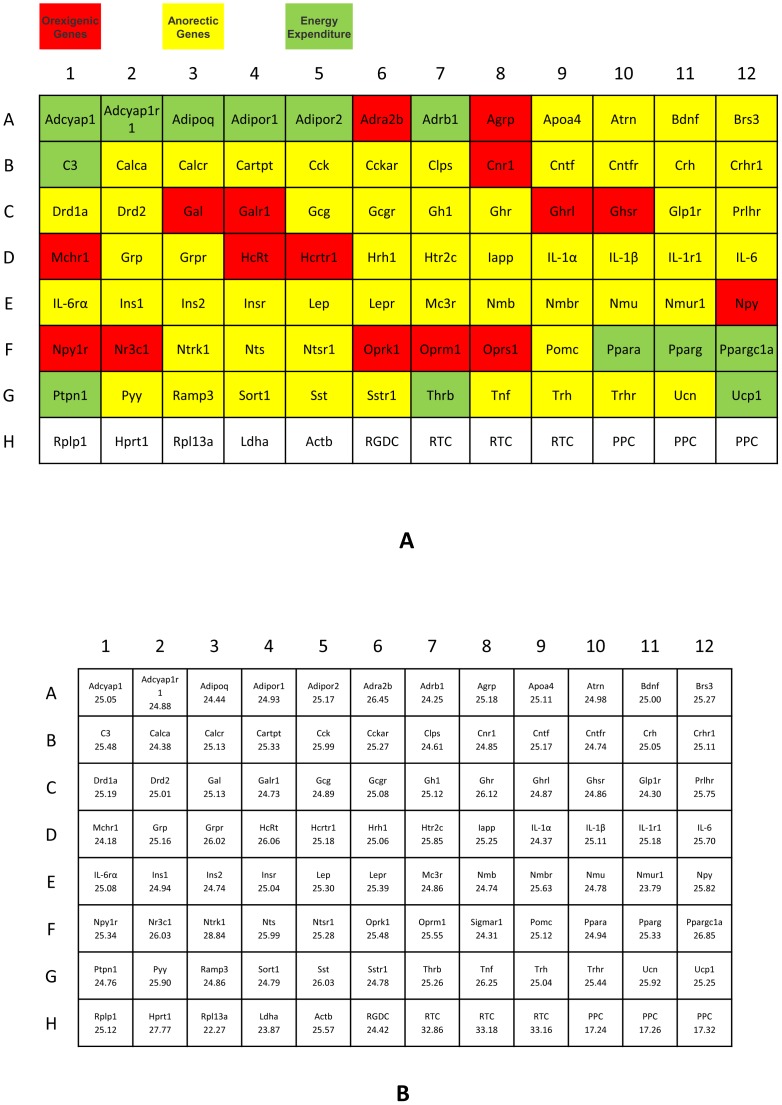
Rat obesity PCR Array. (A) Functional gene grouping in 3 colors: orexigenic genes in red, anorectic genes in yellow, and genes involved in energy expenditure in green. (B) The C_T_ value of quality control used rat genomic DNA.

Constitutive gene expression profiling was performed using the RT^2^ Profiler™ PCR array (SABiosciences) related to obesity signal transduction based on manufacturer’s instructions. The rat genomic DNA was used first to test the array for quality control. The gene array profiled the expression of 84 genes including orexigenic genes, anorectic genes, and genes related to energy expenditure (Table 1 and Figure 1A, PARN-017A-12, RT^2^ Profiler™ PCR Array Rat Obesity). In addition, the array included the controls for human genomic DNA contamination, reverse transcription, positive PCR control, and 5 housekeeping genes [ribosomal protein large P1 (Rplp1), hypoxanthine phosphoribosyltransferase 1 (Hprt1), ribosomal protein L13A (Rpl13a), lactate dehydrogenase A (Ldha), and actin beta (Actb) (Tables S1, S2, and S3). These 5 housekeeping genes were used in normalizing the relative gene expression for data analysis. Briefly, an aliquot of 102 µL diluted cDNA synthesis reaction was mixed with an experimental cocktail containing 1,275 µL 2× RT^2^ SYBR® green/fluorescein qPCR master mix (SABiosciences) and 1,173 µL Mili-Q water (18.3 M, pH 6.8) to form the PCR master mixture. An aliquot of 25 µL of the mixture (a total of 9.0 ng cDNA) was added to each well of the 96-well PCR array. Real-time PCR was performed using a two-step cycling program on an ABI PRISM 7000 System (Applied Biosystems) under the following conditions: 10 min at 95°C (cycle 1) followed by 40 cycles of 15 s at 95°C and 1 min at 60°C. SYBR® green fluorescence was detected and recorded. The threshold cycle (C_T_) above the background for each reaction was calculated. The quality control using rat genomic DNA demonstrated all the targets on this PCR array were detectable as expected and ready for use (Figure 1B).

**Table 1:** The symbol and description of genes in the PCR array.

Position	Symbol	Description	Gene Name
A01	Adcyap1	Adenylate cyclase activating polypeptide 1	Pacap
A02	Adcyap1r1	Adenylate cyclase activating polypeptide 1 receptor 1	PACAP-R1A, PACAPR1, PACAPR1A
A03	Adipoq	Adiponectin, C1Q and collagen domain containing	Acdc, Acrp30
A04	Adipor1	Adiponectin receptor 1	–
A05	Adipor2	Adiponectin receptor 2	–
A06	Adra2b	Adrenergic, alpha-2B-, receptor	–
A07	Adrb1	Adrenergic, beta-1-, receptor	B1AR, RATB1AR
A08	Agrp	Agouti related protein homolog (mouse)	–
A09	Apoa4	Apolipoprotein A-IV	Apo-AIV, ApoA-IV, apoAIV
A10	Atrn	Attractin	–
A11	Bdnf	Brain-derived neurotrophic factor	MGC105254
A12	Brs3	Bombesin-like receptor 3	–
B01	C3	Complement component 3	–
B02	Calca	Calcitonin-related polypeptide alpha	CAL6, CGRP, Cal1, Calc, RATCAL6, calcitonin
B03	Calcr	Calcitonin receptor	–
B04	Cartpt	CART prepropeptide	Cart
B05	Cck	Cholecystokinin	–
B06	Cckar	Cholecystokinin A receptor	Cck-ar
B07	Clps	Colipase, pancreatic	COLQ
B08	Cnr1	Cannabinoid receptor 1 (brain)	SKR6R
B09	Cntf	Ciliary neurotrophic factor	–
B10	Cntfr	Ciliary neurotrophic factor receptor	–
B11	Crh	Corticotropin releasing hormone	CRF
B12	Crhr1	Corticotropin releasing hormone receptor 1	–
C01	Drd1a	Dopamine receptor D1A	D1a, Drd-1, Drd1
C02	Drd2	Dopamine receptor D2	–
C03	Gal	Galanin prepropeptide	Galn
C04	Galr1	Galanin receptor 1	Galnr1
C05	Gcg	Glucagon	GLP-1
C06	Gcgr	Glucagon receptor	MGC93090
C07	Gh1	Growth hormone 1	Gh, RNGHGP
C08	Ghr	Growth hormone receptor	GHR, BP, MGC124963, MGC156665
C09	Ghrl	Ghrelin/obestatin prepropeptide	–
C10	Ghsr	Growth hormone secretagogue receptor	–
C11	Glp1r	Glucagon-like peptide 1 receptor	Glip, RATGL1RCP
C12	Prlhr	Prolactin releasing hormone receptor	Gpr10, Uhr-1
D01	Mchr1	Melanin-concentrating hormone receptor 1	Gpr24, Mch-1r, Slc1
D02	Grp	Gastrin releasing peptide	–
D03	Grpr	Gastrin releasing peptide receptor	–
D04	HcRt	Hypocretin	orexin-A
D05	Hcrtr1	Hypocretin (orexin) receptor 1	Hctr1
D06	Hrh1	Histamine receptor H 1	Hisr
D07	Htr2c	5-hydroxytryptamine (serotonin) receptor 2C	5-HT2C, 5-HTR2C, 5HT-1C
D08	Iapp	Islet amyloid polypeptide	–
D09	IL-1α	Interleukin 1 alpha	IL-1 alpha
D10	IL-1β	Interleukin 1 beta	–
D11	IL-1r1	Interleukin 1 receptor, type I	–
D12	IL-6	Interleukin 6	ILg6, Ifnb2
E01	IL-6rα	Interleukin 6 receptor	IL6R1, Il6ra, Il6r
E02	Ins1	Insulin 1	–
E03	Ins2	Insulin 2	–
E04	Insr	Insulin receptor	–
E05	Lep	Leptin	OB, obese
E06	Lepr	Leptin receptor	Fa
E07	Mc3r	Melanocortin 3 receptor	–
E08	Nmb	Neuromedin B	RGD1562710
E09	Nmbr	Neuromedin B receptor	NMB-R
E10	Nmu	Neuromedin U	–
E11	Nmur1	Neuromedin U receptor 1	Gpr66
E12	Npy	Neuropeptide Y	NPY02, RATNPY, RATNPY02
F01	Npy1r	Neuropeptide Y receptor Y1	MGC109393, NPY-1
F02	Nr3c1	Nuclear receptor subfamily 3, group C, member 1	GR, Gcr, Grl
F03	Ntrk1	Neurotrophic tyrosine kinase, receptor, type 1	Trk
F04	Nts	Neurotensin	–
F05	Ntsr1	Neurotensin receptor 1	Ntsr
F06	Oprk1	Opioid receptor, kappa 1	–
F07	Oprm1	Opioid receptor, mu 1	MORA, Oprm, Oprrm1
F08	Sigmar1	Sigma non-opioid intracellular receptor 1	Oprs1
F09	Pomc	Proopiomelanocortin	Pomc1, Pomc2
F10	Ppara	Peroxisome proliferator activated receptor alpha	PPAR
F11	Pparg	Peroxisome proliferator-activated receptor gamma	–
F12	Ppargc1a	Peroxisome proliferator-activated receptor gamma, coactivator 1 alpha	Ppargc1
G01	Ptpn1	Protein tyrosine phosphatase, non-receptor type 1	MGC93562, Ptp
G02	Pyy	Peptide YY (mapped)	GHYY, RATGHYY, Yy, peptide-YY
G03	Ramp3	Receptor (G protein-coupled) activity modifying protein 3	–
G04	Sort1	Sortilin 1	Nt3, Nts3
G05	Sst	Somatostatin	SS-14, SS-28, Smst
G06	Sstr1	Somatostatin receptor 1	Gpcrrna
G07	Thrb	Thyroid hormone receptor beta	C-erba-beta, ERBA2, Nr1a2, RATT3REC, T3rec, TRbeta
G08	Tnf	Tumor necrosis factor (TNF superfamily, member 2)	MGC124630, RATTNF, TNF-alpha, Tnfa
G09	Trh	Thyrotropin releasing hormone	THR, TRH01
G10	Trhr	Thyrotropin releasing hormone receptor	–
G11	Ucn	Urocortin	–
G12	Ucp1	Uncoupling protein 1 (mitochondrial, proton carrier)	MGC108736, Ucp, Ucpa, Uncp
H01	Rplp1	Ribosomal protein, large, P1	MGC72935
H02	Hprt1	Hypoxanthine phosphoribosyltransferase 1	Hgprtase, Hprt, MGC112554
H03	Rpl13a	Ribosomal protein L13A	–
H04	Ldha	Lactate dehydrogenase A	Ldh1
H05	Actb	Actin, beta	Actx
H06	RGDC	Rat Genomic DNA Contamination	RGDC
H07	RTC	Reverse Transcription Control	RTC
H08	RTC	Reverse Transcription Control	RTC
H09	RTC	Reverse Transcription Control	RTC
H10	PPC	Positive PCR Control	PPC
H11	PPC	Positive PCR Control	PPC
H12	PPC	Positive PCR Control	PPC

### IL-1β and IL-6 Analysis

Serum samples were collected from rats (11 months old) at end of the experiment. Serum proinflammatory cytokines IL-1β and IL-6 were analyzed by Bio-Plex™ Rat Cytokine Panel (Bio-Rad Laboratories, Inc., Hercules, CA, USA) with Luminex 100 Analyzer (Luminex Corporation, Austin, TX) following the manufacturer’s instruction.

### Western Blot

The individual rat liver tissue in the control, HF, and HF+GTP groups (n = 9/group, the same samples analyzed in PCR array) was resuspended in a Radio Immuno Preciptation Assay (RIPA) lysis buffer. The samples were homogenized by sonication on ice with a sonic dismembrator (model 100, Fisher, Pittsburgh, PA). Similar to the pooling strategy used in PCR array analysis, three pooled samples were obtained in each of the control, HF, and HF+GTP groups in which an aliquot of 300 µg protein from 3 individual samples in each group was added together to form a pooled sample. Protein concentrations were measured using the Bradford protein assay (Bio-Rad Life Science, Hercules, CA). A total of 90 µg of protein from each of the pooled samples in the control, HF, and HF+GTP groups were separated by 12% SDS-polyacrylamide gel electrophoresis and then transferred to polyvinylidene fluoride (PVDF) membranes. The immobilized proteins were then incubated overnight at 4°C in blocking buffer containing 3% nonfat dry milk in 1× phosphate buffered saline (PBS) and 0.1% Tween 20 (1× PBST). After blocking, the membranes were probed with the primary antibody (anti-SOD1 or anti-COMT, Santa Cruz Biotechnology, Santa Cruz, CA) for 1 h. Antibody binding was detected with donkey anti-rabbit IgG-HRP for 1 h at room temperature. After a brief incubation with enhanced chemiluminescence, the signals on membranes were exposed to X-ray films. Relative densitometric digital analysis of protein bands were determined using Quantity One software (Bio-Rad) and normalized by the intensity of the housekeeping gene (α-Tubulin, Abcam, Cambridge, MA) for each sample.

### Statistical Analyses

Data are expressed as mean ± standard error (SE), unless stated. Data of body weight were analyzed by one-way analysis of variance (ANOVA) with repeated measures followed by Fisher protected least significant difference (Fisher’s LSD) post-hoc tests to evaluate the effects of time and treatment. Data from RT-PCR were quantified by the ABI sequence detection software and normalized by 5 housekeeping genes (endogenous control). ΔC_T_ was defined as the value of subtracting the C_T_ value of endogenous control from the C_T_ value of the target messenger RNA (mRNA). The fold change among groups could be obtained by ΔΔC_T_. *P* value less than 0.05 based on *t*-test was used as the criteria to determine the differentially expressed genes. Data of serum biomarkers were analyzed by one-way ANOVA followed by Fisher’s LSD post-hoc tests to evaluate the treatment effects. For the protein expression levels among the control, HF, and HF+GTP groups, one-way ANOVA and Tukey’s post hoc tests were used to compare densitometric intensity of individual samples between groups. All analyses were performed using SPSS software (SPSS, Inc., Chicago, IL, USA) and differences with P<0.05 were considered statistically significant.

## Results

### Food Intake and Body Weight

**Figure 2:**
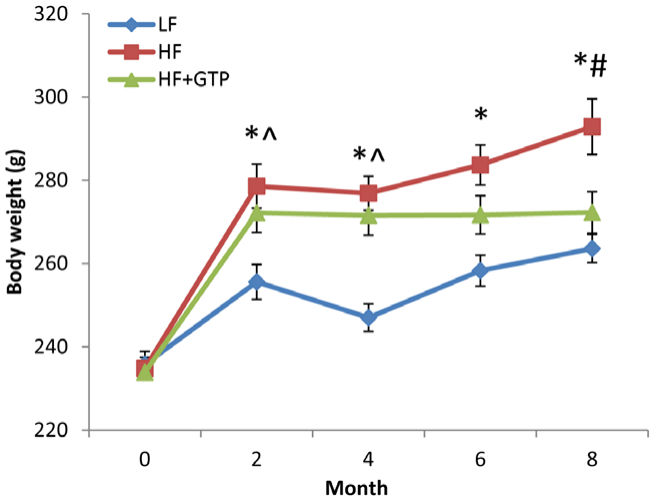
Body weight in female rats supplemented with green tea polyphenols (GTP) in drinking water for 4 months. Body weight (g) of the control, HF, and HF+GTP treated rats at 0–8 months. Values are mean (n = 10–12) with their standard error (SE) represented by vertical bars. * P<0.05 between the control and HF groups; # P<0.05 between the HF and HF+GTP groups; ∧P<0.01 between the control and HF+GTP groups.

Throughout the study, the average food consumption was similar among the treatment groups (13.9±1.6, 12.8±0.6, and 12.1±1.1 g/day for the control, HF, and HF+GTP groups, respectively). The caloric intakes were 53.5±6.2, 60.5±2.8, and 57.2±5.2 kcal/day for the control, HF, and HF+GTP groups, respectively. No difference in initial body weight was seen among all treatment groups (Figure 2). The HF-treated group had significantly greater body weight as compared to the control group at 4 months. At the end of study, supplementation of GTP in drinking water (the HF+GTP group) significantly prevented weight gain compared to the high fat only diet (the HF group) (P<0.05, Figure 2) with an order of body weight: the HF group> the HF+GTP group  =  the control group.

### Gene Expression Alterations

**Figure 3:**
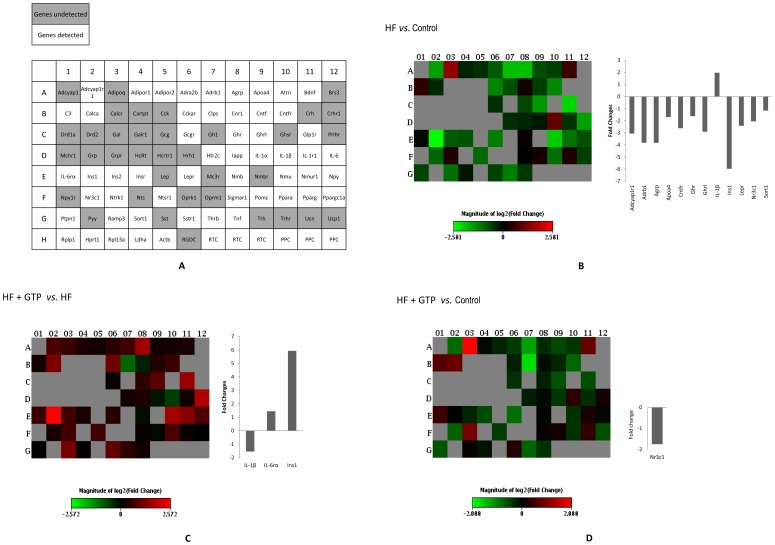
The changes of obesity-related genes among the control, HF, and HF+GTP groups. (A) Representative PCR array gene tables of undetected genes and detected genes. (B) The heat map demonstrating fold regulation expression data between the HF group and the control group. Genes with significant differences between two groups are shown in the histogram. (C) The heat map demonstrating fold regulation expression data between the HF+GTP group and the HF group. Genes with significant differences between two groups are shown in the histogram. (D) The heat map demonstrating fold regulation expression data between the HF+GTP group and the control group. Genes with significant differences between two groups are shown in the histogram.

The triplicate samples from each group produced reproducible results. A total of 35 genes (as measured by C_T_ value) were assigned as undetectable in all groups, leaving 49 genes detected (Tables S1, S2, and S3 and Figure 3A). Relative to the control group, the HF group significantly decreased expression of 11 genes [adenylate cyclase activating polypeptide 1 (pituitary) receptor 1 (Adcyap1r1), adrenergic beta-1-receptor (Adrb1), agouti related protein homolog (Agrp), apolipoprotein A-IV (Apoa4), ciliary neurotrophic factor receptor (Cntfr), growth hormone receptor (Ghr), ghrelin/obestatin prepropeptide (Ghrl), insulin 1 (Ins1), leptin receptor (Lepr), nuclear receptor subfamily 3 group C member 1 (Nr3c1), and sortilin 1 (Sort1)] and increased that of interleukin 1 beta (IL-1β) (P<0.05, Figure 3B). Supplementation of GTP to drinking water (the HF+GTP group) significantly reverted the gene expression levels of IL-1β and Ins1 compared to those of the non-supplemented group (the HF group) (P<0.05, Figure 3C). In addition, the gene expression of interleukin 6 receptor alpha (IL-6rα) was significantly increased in the HF+GTP group compared to the HF group (P<0.05, Figure 3C). Finally, Nr3c1 was the only gene showing significantly down-regulation in the HF+GTP group as compared to the control group (P<0.05, Figure 3D).

### Blood Analysis

**Figure 4:**
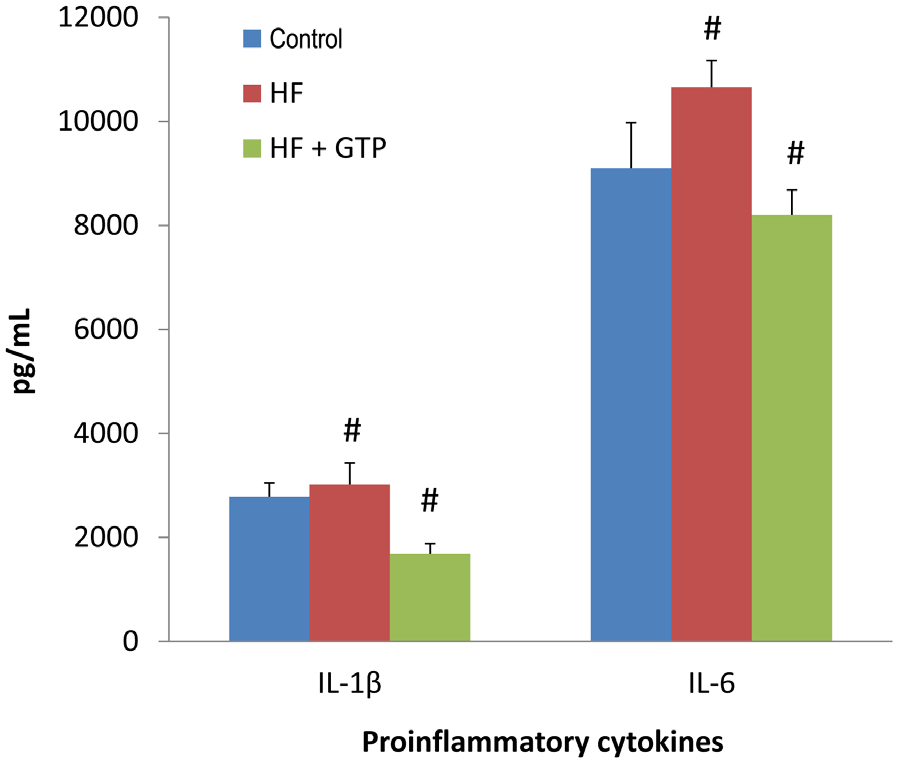
Proinflammatory cytokines changes among the control, HF, and HF+GTP groups. Results are expressed as mean±SE. # P<0.05 between the HF and HF+GTP groups.

The serum levels of IL-1β and IL-6 in the control, HF, and HF+GTP groups are shown in Figure 4. In comparison to the control group or the HF+GTP group, the HF group had the highest serum IL-1β and IL-6 concentrations while the HF+GTP group had the lowest serum IL-1β and IL-6 concentrations. Compared to the HF group, 4-month GTP supplementation to the HF diet (the HF+GTP group) caused a significant decrease of serum proinflammatory cytokines including IL-1β and IL-6 (P<0.05, Figure 4).

### Western Blot Analyses of SOD1 and COMT Protein Expression

**Figure 5:**
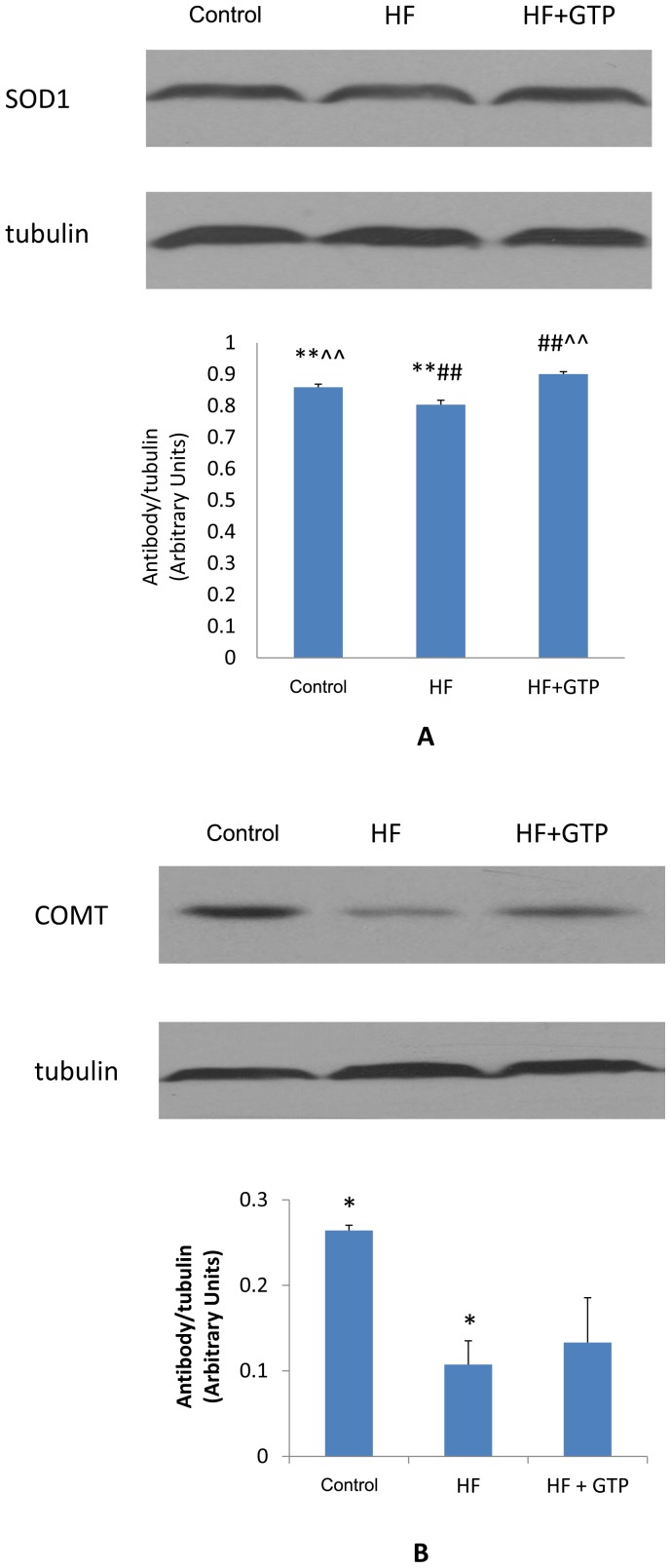
Western blot analyses of protein expression in the control, HF, and HF+GTP groups. (A) Effects of HF and HF+GTP treatment on the protein expression of SOD1. (B) Effects of HF and HF+GTP treatment on the protein expression of COMT. Blots were also probed for α-tubulin to confirm equal protein loading. The relative protein intensities of SOD1 and COMT were compared with the intensity of α-tubulin. The intensity of each band was quantified using Quantity One software. Data are means±SE, n = 3. The experiments were conducted in triplicate. * P<0.05 between the control and HF groups; ** P<0.01 between the control and HF groups; ## P<0.01 between HF and HF+GTP; ∧∧P<0.01 between the control and HF+GTP groups.

The relative protein expression levels of SOD1 and COMT are shown in Figure 5. SOD1 expression level was significantly decreased in the HF group as compared to the control group (P<0.01, Figure 5A). On the other hand, SOD1 expression level was significantly increased in the HF+GTP group as compared to either HF or control groups (P<0.01, Figure 5A). The expression level of COMT was significantly decreased in the HF group as compared to the control group (P<0.05, Figure 5B).

## Discussion

Numerous studies in genetic, metabolic, hormonal, behavioral, social, and cultural aspects have been conducted to increase our understanding of the cause and treatment of obesity [3], [4], [24], [25], [26]. The physiological and molecular changes observed in this high-fat-induced obese rat model provide a useful insight into the development of obesity in humans. In the study, we also measured % fat mass in the animals using the ImpediVET™ Bioimpedance Spectroscopy device (ImpediMed Limited, Brisbane, Australia) at the beginning and the end of study. There was no difference in % fat mass among the groups at the baseline (21.51±0.97, 21.16±1.10, and 23.61±0.83 for the control, HF, and HF+GTP groups, respectively). At the end of study, the order of % fat mass was the HF group (31.36±0.82) > the HF+GTP group (27.78±0.79)  =  the control group (25.78±0.76). Consistent with other reports of obesity models [27], [28], obese rats by a HF diet resulting in a significant increase in % fat mass indicates the difference in body weight was due to mainly the increased fat mass.

In the present study, we have shown that a number of genes involved in obesity changed in HF-treated rats, which further demonstrated the usefulness of this rat model. In addition, our results showed the reduced and controlled weight due to the anti-obesity activity of GTP supplementation. This beneficial effect could be a result of GTP-induced alteration of obesity related genes, anti-inflammation activity, anti-oxidative stress capacity, as well as estrogen-like actions which in turn regulate appetite, metabolism, or absorption of calories.

A total of 12 obesity-related genes showed significant changes in the HF-treated obese rats as compared to those of the control. Among these, Adcyap1r1, Adrb1, Agrp, Apoa4, Cntfr, Ghr, Ghrl, Ins1, Lepr, Nr3c1, and Sort1 decreased significantly while IL-1β increased significantly in the HF group in comparison with those in the control group. There is considerable evidence that these genes play three critical roles in the pathogenesis of obesity, including orexigenic function, anorectic function, and energy expenditure.

Agrp, Ghrl, and Nr3c1 are classified as orexigenic genes. Agrp is one of the most potent and long-lasting of appetite stimulators [29], [30]. The appetite stimulating effects of Agrp are inhibited by the hormone leptin (higher in the HF group than the control group, data not shown) and activated by the hormone ghrelin (Ghrl, lower in the HF group than the control group). Ghrelin (Ghrl) is the endogenous ligand for the growth hormone secretagogue receptor (GHSR) for maintaining growth hormone (GH) release and energy homeostasis [31]. Previous studies have demonstrated the decreased level of circulating ghrelin in humans with obesity [32], [33]. Similarly, Nr3c1, the glucocorticoid receptor gene, has been reported to be involved in hyperinsulinaemia, fat deposition, and inflammatory responses associated with obesity [34]. Nr3c1 mRNA levels have also been reported to be significantly decreased in obesity [35], [36].

Apoa4, Cntf, Ghr, IL-1β, Ins1, Lepr, and Sort are anorectic genes. Apolipoprotein (Apoa4 or Apo A-IV), activated through a dietary fat-elicited satiety signal [37], [38], is involved in various activities, such as antioxidant activity, cholesterol transporter activity, lipid binding, phosphatidylcholine binding [39], [40], [41]. Therefore, the decreased gene expression level of Apoa4 in the HF group could contribute to diet-induced obesity. Cntfr can mimic the biological actions of leptin while overcoming “leptin resistance” [42]. Ghr has profound effects on adipogenesis, lipogenesis and lipolysis by inducing alterations in the central nervous system [43], [44]. Studies have suggested that decreased Ghr availability could result in obesity [45], [46]. Insulin, which plays important roles in regulating carbohydrate and fat metabolism in the body, is commonly deficient or resistant in obese patients [47]. The decreased gene expression level of Ins1 in the HF group further supports this rat obese model. Leptin is one of the most important adipose derived hormones, which secretes into the bloodstream and is transported to the brain, where it stimulates or inhibits release of several neurotransmitters [48], [49], [50]. In line with our findings, lepr and subsequent signaling events have been reported to be down-regulated in the liver of dietary-induced obese rats [51]. Finally, sortilin exhibits the strongest association with serum lipoproteins by degrading nascent VLDL particles [52], [53].

Adcyap1r1 and Adrb1 are the two genes that relate to energy expenditure. Adcyap1r1 can influence the control of energy homeostasis and has remarkable anti-inflammation action [54], [55]. Adrb1 induces lipolysis in adipose tissue [56]. In all, the altered expression levels of 12 obesity genes in the HF group, as compared to the control group, suggest a qualified obese model, which could result in reverted obesity via regulating the expression of obesity-related genes. The obesity model used in our study may more closely simulate the onset of obesity in humans, rather than the genetic alteration or mutation induced obesity models.

The findings that GTP supplementation into the drinking water reversed the expression levels of 11/12 genes (except Nr3c1) in obese rats corroborates the anti-obesity role of GTP. For instance, our findings of increased Adcyap1r1 and Adrb1 after GTP treatment are in agreement with studies, demonstrating that green tea increases energy expenditure [57], [58], [59], and has proved effective in reducing body fat partially mediated via Adrb1 activation [60].

Chronic systemic inflammation directly contributes to the development of obesity [5], [54]–[57]. For instance, overweight and obese women generally have elevated serum levels of inflammation cytokines, such as IL-6 and TNF-α [61], [62], [63]. Therefore, suppressing chronic inflammation may be a good strategy to prevent and/or treat obesity. The observations that the HF group increased IL-1β gene expression agrees with Matsuki *et al.* that IL-1β is harmful for the energy homeostasis of the body through its role in lipid metabolism by regulating insulin levels [64]. GTP supplementation suppressed the chronic inflammation due to a high-fat diet in the present study, as shown by decreased IL-1β gene expression and circulating IL-1β and IL-6 levels, as well as increased IL-6rα and Inl1 gene expression. However, although higher expression levels of IL-1β and IL-6 in the serum from rats of the HF group were observed as compared to those of the control, these differences did not reach statistical significances. This might due to the large variations within the groups. Therefore, future studies using a larger sample size are warranted.

One of the other most widely recognized characteristics of obesity is the increased oxidative stress [65]. GTP has shown its anti-oxidant activities, stemming from its ability to scavenge reactive oxygen species [66]. In the present study, GTP supplementation increased SOD1 expression (an indicator of antioxidant capacity) in obese rats. Our results are consistent with the anti-obesity role of GTP by increasing antioxidant capacity and/or decreasing oxidative stress damage, which is commonly observed in obesity [20], [24], [67], [68], [69].

Although there was no statistically significant difference in COMT protein expression between the HF+GTP group and the HF group due to the large variation, this restoration of COMT made no statistical significance between HF+GTP and control groups. COMT activity is important in fat regulation, since preadipocyte proliferation and differentiation can be inhibited by 2-methoxyestradiol, the synthesis of which is catalyzed by COMT [70]. Weight loss results in a reduction in circulating estrogens [71], [72]. Therefore, the restoration of COMT expression observed after GTP treatment would inhibit the activation of estrogen by catalyzing the methylation of catechol estrogens to less active methoxy estrogens (e.g., 2-methoxyestradiol). Moreover, COMT has been shown to be involved in reward-motivated behaviors such as development of diet-induced obesity through metabolizing dopamine [73]. Taken together, the GTP-induced weight loss may possibly and partially result from increased COMT through its regulation of estrogen and appetite.

Among all the 84 genes related to obesity, a total of 35 genes were not detected in all 3 groups of our rat liver samples. There might be reasons such as genes are not (or very weakly) expressed in the liver tissues, for example Brs3, which is normally expressed in the brain, kidney, and testis. Calcr is normally expressed in the brain and kidney. Cartpt can be detected in the brain, spinal cord, prostate, and testis but not in the liver [74]; and the detection sensitivity of gene array is limited. On the other hand, due to the mixture of cell types in tissue samples, the magnitude of expression changes of some genes might not be detected.

Studies including ours have demonstrated the effects of green tea on weight loss. Possible mechanisms behind this may result from the observation of lower serum and LDL cholesterol, increased HDL cholesterol, and lower serum glucose [75], [76], [77]; the increase in fat mobilization and oxidation [15], [16], [78], [79]; the increase in energy expenditure [80] and appetite suppression [81], [82]; as well as the reduction in the absorption of nutrients [83] as a result of GTP treatment. However, there are still debates of the overall effects of GTP that show some inconsistent results [84], [85], [86], [87]. For instance, several studies have failed to show differences in assessed subjective appetite or energy intake after green tea consumption, despite favorable outcomes for body weight and body fat [6], [12], [88]. These might partially due to the variation and/or the sensibility of detecting small changes in dietary intake, which are hard to be accurately measured with available techniques. As an outcome, this minor imbalance between energy intake and energy expenditure over years may lead to severe obesity. In fact, in lines with these findings, the correlation between the changes of orexigenic genes and the behavior of appetite was not observed in our current study. Collectively, this could lead to the difficulty in assessing the etiology of obesity as well as the protective effects of GTP supplement. Nevertheless, our study firstly demonstrated the changes of anorectic and/or orexigenic genes in the obese rat, while GTP supplement could reverse the effects of these genes. The study strategy conducted in the present study could provide an additional concept in assessing not only the etiologic mechanisms of obesity but the beneficial effects of GTP. However, this study is limited in lacking a control+GTP group which would provide additional evidences to demonstrate the beneficial effects of GTP supplement. In addition, the study using male mice could be helpful in expanding the findings of the present study to better understand the protective effects of GTP supplement in obesity.

In summary, our data demonstrated that GTP supplementation has potent effects on body weight in obese middle-aged female rats through regulating obesity-related genes, anti-inflammation activity, anti-oxidative stress capacity, as well as estrogen-associated actions. We believe that our study presents a critical first step towards evaluating the effects of green tea consumption in obese middle-aged women. Translational research based on findings from animal observations to investigate possible therapeutic efficacy of GTP on obese women will be worthy in a future study.

## Supporting Information

Table S1 **Effects of HF on the expression levels of obesity related genes as compared to the control.**
(XLS)

Table S2 **Effects of HF+GTP on the expression levels of obesity related genes as compared to HF.**
(XLS)

Table S3 **Effects of HF+GTP on the expression levels of obesity related genes as compared to the control.**
(XLS)

## References

[1] Weight-control Information Network website.. http://win.niddk.nih.gov/statistics/#overweight.

[2] Calle EE, Rodriguez C, Walker-Thurmond K, Thun MJ (2003). Overweight, obesity, and mortality from cancer in a prospectively studied cohort of U.S. adults.. N Engl J Med.

[3] Must A, Spadano J, Coakley EH, Field AE, Colditz G (1999). The disease burden associated with overweight and obesity.. JAMA.

[4] Spiegelman BM, Flier JS (2001). Obesity and the regulation of energy balance.. Cell.

[5] Rankinen T, Zuberi A, Chagnon YC, Weisnagel SJ, Argyropoulos G (2006). The human obesity gene map: the 2005 update.. Obesity.

[6] Auvichayapat P, Prapochanung M, Tunkamnerdthai O, Sripanidkulchai BO, Auvichayapat N (2008). Effectiveness of green tea on weight reduction in obese Thais: A randomized, controlled trial.. Physiol Behav.

[7] Hsu CH, Tsai TH, Kao YH, Hwang KC, Tseng TY (2008). Effect of green tea extract on obese women: a randomized, double-blind, placebo-controlled clinical trial.. Clin Nutr.

[8] Lonac MC, Richards JC, Schweder MM, Johnson TK, Bell C (2011). Influence of short-term consumption of the caffeine-free, epigallocatechin-3-gallate supplement, Teavigo, on resting metabolism and the thermic effect of feeding.. Obesity (Silver Spring).

[9] Nagao T, Hase T, Tokimitsu I (2007). A green tea extract high in catechins reduces body fat and cardiovascular risks in humans.. Obesity (Silver Spring).

[10] Phung OJ, Baker WL, Matthews LJ, Lanosa M, Thorne A (2010). Effect of green tea catechins with or without caffeine on anthropometric measures: a systematic review and meta-analysis.. Am J Clin Nutr.

[11] Stendell-Hollis NR, Thomson CA, Thompson PA, Bea JW, Cussler EC (2010). Green tea improves metabolic biomarkers, not weight or body composition: a pilot study in overweight breast cancer survivors.. J Hum Nutr Diet.

[12] Wang H, Wen Y, Du Y, Yan X, Guo H (2010). Effects of catechin enriched green tea on body composition.. Obesity (Silver Spring).

[13] Westerterp-Plantenga MS (2010). Green tea catechins, caffeine and body-weight regulation.. Physiol Behav.

[14] Basu A, Sanchez K, Leyva MJ, Wu M, Betts NM (2010). Green tea supplementation affects body weight, lipids, and lipid peroxidation in obese subjects with metabolic syndrome.. J Am Coll Nutr.

[15] Boschmann M, Thielecke F (2007). The effects of epigallocatechin-3-gallate on thermogenesis and fat oxidation in obese men: a pilot study.. J Am Coll Nutr.

[16] Rains TM, Agarwal S, Maki KC (2011). Antiobesity effects of green tea catechins: a mechanistic review.. J Nutr Biochem.

[17] Shen CL, Yeh JK, Samathanam C, Cao JJ, Stoecker BJ (2011). Green tea polyphenols attenuate deterioration of bone microarchitecture in female rats with systemic chronic inflammation.. Osteoporos Int.

[18] Shen CL, Yeh JK, Cao JJ, Tatum OL, Dagda RY (2010). Green tea polyphenols mitigate bone loss of female rats in a chronic inflammation-induced bone loss model.. J Nutr Biochem.

[19] Shen CL, Yeh JK, Stoecker BJ, Chyu MC, Wang JS (2009). Green tea polyphenols mitigate deterioration of bone microarchitecture in middle-aged female rats.. Bone.

[20] Shao C, Chen L, Lu C, Shen CL, Gao W (2011). A gel-based proteomic analysis of the effects of green tea polyphenols on ovariectomized rats.. Nutrition.

[21] Shen CL, Yeh JK, Cao JJ, Chyu MC, Wang JS (2011). Green tea and bone health: Evidence from laboratory studies.. Pharmacol Res.

[22] Shen CL, Wang P, Guerrieri J, Yeh JK, Wang JS (2008). Protective effect of green tea polyphenols on bone loss in middle-aged female rats.. Osteoporos Int.

[23] Shao C, Lu C, Chen L, Koty PP, Cobos E (2011). p53-Dependent anticancer effects of leptomycin B on lung adenocarcinoma.. Cancer Chemother Pharmacol.

[24] Khan NI, Naz L, Yasmeen G (2006). Obesity: an independent risk factor for systemic oxidative stress.. Pak J Pharm Sci.

[25] Jequier E (2002). Leptin signaling, adiposity, and energy balance.. Ann N Y Acad Sci.

[26] Dulloo AG (2002). Biomedicine. A sympathetic defense against obesity.. Science.

[27] Buettner R, Scholmerich J, Bollheimer LC (2007). High-fat diets: modeling the metabolic disorders of human obesity in rodents.. Obesity (Silver Spring).

[28] Dobrian AD, Davies MJ, Schriver SD, Lauterio TJ, Prewitt RL (2001). Oxidative stress in a rat model of obesity-induced hypertension.. Hypertension.

[29] Ollmann MM, Wilson BD, Yang YK, Kerns JA, Chen Y (1997). Antagonism of central melanocortin receptors in vitro and in vivo by agouti-related protein.. Science.

[30] Shutter JR, Graham M, Kinsey AC, Scully S, Luthy R (1997). Hypothalamic expression of ART, a novel gene related to agouti, is up-regulated in obese and diabetic mutant mice.. Genes Dev.

[31] Kojima M, Hosoda H, Date Y, Nakazato M, Matsuo H (1999). Ghrelin is a growth-hormone-releasing acylated peptide from stomach.. Nature.

[32] Tschop M, Weyer C, Tataranni PA, Devanarayan V, Ravussin E (2001). Circulating ghrelin levels are decreased in human obesity.. Diabetes.

[33] Cummings DE, Purnell JQ, Frayo RS, Schmidova K, Wisse BE (2001). A preprandial rise in plasma ghrelin levels suggests a role in meal initiation in humans.. Diabetes.

[34] Rosmond R (2003). Association studies of genetic polymorphisms in central obesity: a critical review.. International journal of obesity.

[35] Boullu-Ciocca S, Paulmyer-Lacroix O, Fina F, Ouafik L, Alessi MC (2003). Expression of the mRNAs coding for the glucocorticoid receptor isoforms in obesity.. Obes Res.

[36] Bronnegard M, Reynisdottir S, Marcus C, Stierna P, Arner P (1995). Effect of glucocorticosteroid treatment on glucocorticoid receptor expression in human adipocytes.. J Clin Endocrinol Metab.

[37] Fujimoto K, Machidori H, Iwakiri R, Yamamoto K, Fujisaki J (1993). Effect of intravenous administration of apolipoprotein A-IV on patterns of feeding, drinking and ambulatory activity of rats.. Brain Res.

[38] Fujimoto K, Fukagawa K, Sakata T, Tso P (1993). Suppression of food intake by apolipoprotein A-IV is mediated through the central nervous system in rats.. J Clin Invest.

[39] Wai-man RW, Stephens JW, Acharya J, Hurel SJ, Humphries SE (2004). The APOA4 T347S variant is associated with reduced plasma TAOS in subjects with diabetes mellitus and cardiovascular disease.. Journal of lipid research.

[40] Wong WMR, Gerry AB, Putt W, Roberts JL, Weinberg RB (2007). Common variants of apolipoprotein A-IV differ in their ability to inhibit low density lipoprotein oxidation.. Atherosclerosis.

[41] Navab M, Hama SY, Ready ST, Ng CJ, Van Lenten BJ (2002). Oxidized lipids as mediators of coronary heart disease.. Current opinion in lipidology.

[42] Matthews VB, Febbraio MA (2008). CNTF: a target therapeutic for obesity-related metabolic disease?. J Mol Med (Berl).

[43] Nam S, Lobie P (2000). The mechanism of effect of growth hormone on preadipocyte and adipocyte function.. obesity reviews.

[44] Ballesteros M, Leung KC, Ross RJM, Iismaa TP, Ho KKY (2000). Distribution and abundance of messenger ribonucleic acid for growth hormone receptor isoforms in human tissues.. Journal of Clinical Endocrinology & Metabolism.

[45] Egecioglu E, Bjursell M, Ljungberg A, Dickson SL, Kopchick JJ (2006). Growth hormone receptor deficiency results in blunted ghrelin feeding response, obesity, and hypolipidemia in mice.. American Journal of Physiology-Endocrinology And Metabolism.

[46] Erman A, Wabitsch M, Goodyer C (2011). Human growth hormone receptor (GHR) expression in obesity: II. Regulation of the human GHR gene by obesity-related factors. International journal of obesity..

[47] Kahn BB, Flier JS (2000). Obesity and insulin resistance.. Journal of Clinical Investigation.

[48] Brennan AM, Mantzoros CS (2006). Drug Insight: the role of leptin in human physiology and pathophysiology–emerging clinical applications.. Nat Clin Pract Endocrinol Metab.

[49] Houseknecht KL, Baile CA, Matteri RL, Spurlock ME (1998). The biology of leptin: a review.. J Anim Sci.

[50] Houseknecht KL, Portocarrero CP (1998). Leptin and its receptors: regulators of whole-body energy homeostasis.. Domest Anim Endocrinol.

[51] Brabant G, Muller G, Horn R, Anderwald C, Roden M (2005). Hepatic leptin signaling in obesity.. FASEB J.

[52] Musunuru K, Strong A, Frank-Kamenetsky M, Lee NE, Ahfeldt T (2010). From noncoding variant to phenotype via SORT1 at the 1p13 cholesterol locus.. Nature.

[53] Teslovich TM, Musunuru K, Smith AV, Edmondson AC, Stylianou IM (2010). Biological, clinical and population relevance of 95 loci for blood lipids.. Nature.

[54] Elekes K, Sandor K, Moricz A, Kereskai L, Kemeny A (2011). Pituitary adenylate cyclase-activating polypeptide plays an anti-inflammatory role in endotoxin-induced airway inflammation: in vivo study with gene-deleted mice.. Peptides.

[55] Garfield AS, Lam DD, Marston OJ, Przydzial MJ, Heisler LK (2009). Role of central melanocortin pathways in energy homeostasis.. Trends in Endocrinology & Metabolism.

[56] Gjesing A, Andersen G, Albrechtsen A, Glümer C, Borch Johnsen K (2007). Studies of associations between the Arg389Gly polymorphism of the 1 adrenergic receptor gene (ADRB1) and hypertension and obesity in 7677 Danish white subjects.. Diabetic medicine.

[57] Rumpler W, Seale J, Clevidence B, Judd J, Wiley E (2001). Oolong tea increases metabolic rate and fat oxidation in men.. J Nutr.

[58] Dulloo AG, Duret C, Rohrer D, Girardier L, Mensi N (1999). Efficacy of a green tea extract rich in catechin polyphenols and caffeine in increasing 24-h energy expenditure and fat oxidation in humans.. Am J Clin Nutr.

[59] Chantre P, Lairon D (2002). Recent findings of green tea extract AR25 (Exolise) and its activity for the treatment of obesity.. Phytomedicine.

[60] Choo JJ (2003). Green tea reduces body fat accretion caused by high-fat diet in rats through beta-adrenoceptor activation of thermogenesis in brown adipose tissue.. J Nutr Biochem.

[61] Yudkin JS, Stehouwer C, Emeis J, Coppack S (1999). C-reactive protein in healthy subjects: associations with obesity, insulin resistance, and endothelial dysfunction: a potential role for cytokines originating from adipose tissue?. Arteriosclerosis, Thrombosis, and Vascular Biology.

[62] Festa A, D'Agostino Jr R, Williams K, Karter A, Mayer-Davis E (2001). The relation of body fat mass and distribution to markers of chronic inflammation.. International journal of obesity.

[63] Cottam DR, Mattar SG, Barinas-Mitchell E, Eid G, Kuller L (2004). The chronic inflammatory hypothesis for the morbidity associated with morbid obesity: implications and effects of weight loss.. Obes Surg.

[64] Matsuki T, Horai R, Sudo K, Iwakura Y (2003). IL-1 plays an important role in lipid metabolism by regulating insulin levels under physiological conditions.. J Exp Med.

[65] Bondia-Pons I, Ryan L, Martinez JA (2012). Oxidative stress and inflammation interactions in human obesity. J Physiol Biochem.. [Epub ahead of print].

[66] Moon HS, Lee HG, Choi YJ, Kim TG, Cho CS (2007). Proposed mechanisms of (-)-epigallocatechin-3-gallate for anti-obesity.. Chem Biol Interact.

[67] Furukawa S, Fujita T, Shimabukuro M, Iwaki M, Yamada Y (2004). Increased oxidative stress in obesity and its impact on metabolic syndrome.. J Clin Invest.

[68] Keaney JF, Larson MG, Vasan RS, Wilson PW, Lipinska I (2003). Obesity and systemic oxidative stress: clinical correlates of oxidative stress in the Framingham Study.. Arterioscler Thromb Vasc Biol.

[69] Wolfram S, Wang Y, Thielecke F (2006). Anti-obesity effects of green tea: from bedside to bench.. Mol Nutr Food Res.

[70] Pico C, Puigserver P, Oliver P, Palou A (1998). 2-Methoxyestradiol, an endogenous metabolite of 17beta-estradiol, inhibits adipocyte proliferation.. Mol Cell Biochem.

[71] Berrino F, Bellati C, Secreto G, Camerini E, Pala V (2001). Reducing bioavailable sex hormones through a comprehensive change in diet: the diet and androgens (DIANA) randomized trial.. Cancer Epidemiol Biomarkers Prev.

[72] Tchernof A, Nolan A, Sites CK, Ades PA, Poehlman ET (2002). Weight loss reduces C-reactive protein levels in obese postmenopausal women.. Circulation.

[73] Wang SS, Morton LM, Bergen AW, Lan EZ, Chatterjee N (2007). Genetic variation in catechol-O-methyltransferase (COMT) and obesity in the prostate, lung, colorectal, and ovarian (PLCO) cancer screening trial.. Hum Genet.

[74] Genecards website.. http://www.genecards.org.

[75] Mitscher LA, Jung M, Shankel D, Dou JH, Steele L (1997). Chemoprotection: a review of the potential therapeutic antioxidant properties of green tea (Camellia sinensis) and certain of its constituents.. Med Res Rev.

[76] Kao YH, Hiipakka RA, Liao S (2000). Modulation of endocrine systems and food intake by green tea epigallocatechin gallate.. Endocrinology.

[77] Matsumoto N, Ishigaki F, Ishigaki A, Iwashina H, Hara Y (1993). Reduction of blood glucose levels by tea catechin.. Bioscience, biotechnology, and biochemistry.

[78] Klaus S, Pültz S, Thöne-Reineke C, Wolfram S (2005). Epigallocatechin gallate attenuates diet-induced obesity in mice by decreasing energy absorption and increasing fat oxidation.. Int J Obes (Lond).

[79] Murase T, Nagasawa A, Suzuki J, Hase T, Tokimitsu I (2002). Beneficial effects of tea catechins on diet-induced obesity: stimulation of lipid catabolism in the liver.. Int J Obes Relat Metab Disord.

[80] Borchardt RT, Huber JA (1975). Catechol O-methyltransferase. 5. Structure-activity relationships for inhibition by flavonoids.. J Med Chem.

[81] Belza A, Toubro S, Astrup A (2009). The effect of caffeine, green tea and tyrosine on thermogenesis and energy intake.. Eur J Clin Nutr.

[82] Belza A, Frandsen E, Kondrup J (2007). Body fat loss achieved by stimulation of thermogenesis by a combination of bioactive food ingredients: a placebo-controlled, double-blind 8-week intervention in obese subjects.. Int J Obes (Lond).

[83] Hsu TF, Kusumoto A, Abe K, Hosoda K, Kiso Y (2006). Polyphenol-enriched oolong tea increases fecal lipid excretion.. Eur J Clin Nutr.

[84] Chan CC, Koo MW, Ng EH, Tang OS, Yeung WS (2006). Effects of Chinese green tea on weight, and hormonal and biochemical profiles in obese patients with polycystic ovary syndrome–a randomized placebo-controlled trial.. J Soc Gynecol Investig.

[85] Fukino Y, Shimbo M, Aoki N, Okubo T, Iso H (2005). Randomized controlled trial for an effect of green tea consumption on insulin resistance and inflammation markers.. J Nutr Sci Vitaminol (Tokyo).

[86] Diepvens K, Kovacs EM, Vogels N, Westerterp-Plantenga MS (2006). Metabolic effects of green tea and of phases of weight loss.. Physiol Behav.

[87] Hill AM, Coates AM, Buckley JD, Ross R, Thielecke F (2007). Can EGCG reduce abdominal fat in obese subjects?. J Am Coll Nutr.

[88] Maki KC, Reeves MS, Farmer M, Yasunaga K, Matsuo N (2009). Green tea catechin consumption enhances exercise-induced abdominal fat loss in overweight and obese adults.. J Nutr.

